# Supplementary material for: How muscle ageing affects rapid goal-directed movement: mechanistic insights from a simple model
Source: PLoS Comput Biol. 2026 Apr 15;22(4):e1014023. doi: 10.1371/journal.pcbi.1014023 (PMC13082658; doi:10.1371/journal.pcbi.1014023)
Supplement: S1 Appendix — Included are mathematical details of force-length, force-velocity and activation characteristics, as well as analysis of the correlations between RMSE, time to target and coactivation. (PDF) [file pcbi.1014023.s005.pdf]

S1 Appendix

Smooth approximations of Hill-type dynamics

Muscle force was implemented as a Hill-type model of the form

$$F(l, \dot{l}, t) = F_{\max}\left(a(t, u)\left(f_l(l) \cdot f_v(\dot{l})\right) + f_p(l)\right) \tag{6}$$

where f_l, f_v and f_p are underlying force-length, force-velocity and parallel passive force characteristics, respectively, and a is time-dependent activation. These are based on [34,38], which in turn follows [39] and [40].

These underlying models introduce non-smoothness which leads to issues in gradient-based optimisation. Therefore, the models were smoothed or adjusted where necessary to avoid these issues, resulting in the smoothed Hill-type model

$$F_s(l, \dot{l}, t|\mathbf{s}) = F_{\max}\left(a(t|s_1)\left(f_{l,s}(l) \cdot f_{v,s}(\dot{l}|s_2)\right) + f_{p,s}(l|s_3)\right) \tag{7}$$

with the smoothing parameter $s_i > 0$. For the results in this paper, we used $s_1 = 500$ and $s_2 = s_3 = 200$. The following lays out and defines the smoothed functions used in Equation 7

Force-length characteristics

Force-length characteristics were specified following [39] as

$$f_l(\tilde{l}) = e^{-|(\tilde{l}^{b_2}-1)/b_3|^{b_1}}, \quad \tilde{l} >= 0 \tag{8}$$

where $\tilde{l} := l/l_0$ is the normalised muscle length, with l_0 being optimal length. The absolute value function introduces a discontinuity in the derivative; however, if b_1 is even, the absolute value is unnecessary. Therefore, we chose $b_1 = 2$, instead of 1.3 as in [34,38], and removed the absolute value function, yielding

$$f_{l,s}(\tilde{l}) = e^{-\left((\tilde{l}^{b_2}-1)/b_3\right)^2}, \quad \tilde{l} >= 0 \tag{9}$$

Force-velocity characteristics

Otten [39] implements force-velocity as a piecewise function

$$f_v(\tilde{v}) = \begin{cases} f_{ve}(\tilde{v}) := d_2 - (d_2 - 1)\frac{1+\tilde{v}}{1-d_3\tilde{v}} & \tilde{v} \leq 0, \\ f_{vc}(\tilde{v}) := 1 - \frac{\tilde{v}}{1+d_1\tilde{v}} & 0 \leq \tilde{v} \leq 1, \\ 0 & 1 < \tilde{v}, \end{cases} \tag{10}$$

where $v := -\dot{l}/l_0/v_{\max}$ is the normalised contractile velocity. The piecewise nature of the implementation introduces discontinuities, which we removed by sigmoidal smoothing. The sigmoid function

$$\sigma(x|s) := (1 - e^{-sx})^{-1}, \tag{11}$$

with the smoothing parameter $s > 0$, acts as a smooth approximation of a step function. This allows a piecewise function $f(x) = \{f_1(x), x \leq 0; f_2(x), 0 < x\}$ to be approximated as the continuous function $f_s(x|s) = f_1(x)\sigma(-x|s) + f_2(x|s)\sigma(x|s)$, with the approximation fidelity increasing with larger values of s (at the expense of potentially larger first and second derivatives at the piecewise transition).

Using this technique, we first rewrote Equation 10 as

$$f_{v,\mathrm{cont}}(\tilde{v}) = \Big(f_{ve}(\tilde{v})\sigma(-\tilde{v}|s) + f_{vc}(\tilde{v})\sigma(\tilde{v}|s)\Big)\,\sigma(1-\tilde{v}|s) \tag{12}$$

However, as each subfunction f_{ve} and f_{vc} is now evaluated everywhere, they introduce singularities when the denominators are zero. These singularities can be mitigated by augmenting the denominator with a smooth ramp function, which ensures the denominator is everywhere positive. The eccentric contraction function $f_{ve}(\tilde{v})$ is replaced with the augmented function

$$f_{ve,a}(\tilde{v}|s) = d_2 - (d_2-1)\frac{1+\tilde{v}}{1-d_3\tilde{v}(1-\sigma(\tilde{v}|s))}. \tag{13}$$

This ensures that when $\tilde{v} < 0$ (eccentric contraction), $f_{ve,a} \approx f_{ve}$, and when $\tilde{v} = 1/d_3$ (which would otherwise be a singularity), the denominator evaluates to $\sigma(1/d_3|s) \approx 1$. For $\tilde{v} > 0$, the denominator is always positive, and the eccentric term is attenuated overall by sigmoidal smoothing.

The concentric term is similarly augmented to

$$f_{vc,a}(\tilde{v}|s) = 1 - \frac{\tilde{v}}{1+d_1\tilde{v}(1-\sigma(-\tilde{v}|s))} \tag{14}$$

and the fully smoothed approximation of Equation 10 is

$$f_{v,s} = \Big(f_{ve,a}(\tilde{v}|s)\sigma(-\tilde{v}|s) + f_{vc,a}(\tilde{v}|s)\sigma(\tilde{v}|s)\Big)\,\sigma(1-\tilde{v}|s). \tag{15}$$

Parallel Stiffness

Murtola and Richards [34] developed an exponential model of parallel passive stiffness based on [40] and [74],

$$f_p(\tilde{l}) = \begin{cases} c_1(e^{c_2(\tilde{l}-c_3)}-1), & c_3 < \tilde{l}, \\ 0, & \tilde{l} \le c_3, \end{cases} \tag{16}$$

where c_3 is the normalised slack length. Following the procedure outlined above, the piecewise nature of this equation was eliminated through sigmoidal smoothing, generating the smoothed function

$$f_{p,s}(\tilde{l}|s) = c_1(e^{c_2(\tilde{l}-c_3)}-1)\,\sigma(\tilde{l}-c_3|s). \tag{17}$$

First-order activation model

The activation model was first-order, based on [40]. Given the excitation $u(t)$, the instantaneous activation rate is determined *via* the piecewise differential equation

$$\dot{a}(t) = \begin{cases} r_a(t) = \frac{u(t)-a(t)}{A_{\mathrm{on}}(t)}, & a(t) \le u(t), \\ r_d(t) = \frac{u(t)-a(t)}{A_{\mathrm{off}}(t)}, & u(t) < a(t), \end{cases} \tag{18}$$

which specifies activation and deactivation rates using time-varying activation and deactivation parameters

$$A_{\mathrm{on}}(t) = \alpha_a(0.5+1.5a(t)), \quad \text{and} \tag{19}$$

$$A_{\mathrm{off}}(t) = \alpha_d(0.5+1.5a(t))^{-1}. \tag{20}$$

As with other piecewise equations, this was converted to a continuous function with sigmoidal smoothing, yielding the single differential equation

$$\dot{a}(t|s) = r_a(t)\,\sigma(u(t)-a(t)|s) + r_d(t)\,\sigma(a(t)-u(t)|s), \tag{21}$$

Correlations between performance metric, time and coactivation

The performance metric of J_{RMSE} encompasses both a temporal aspect and a spatial aspect. There are two phases to the behaviour: a ballistic phase, achieving rapid movement towards the target, as well as stabilisation phase, where the arm comes to rest at the target.

Here we make the case that the performance metric J_{RMSE} is driven largely by the time to target. We define time to target T_T as as the first timepoint where the muscle length is within 0.5% l_0 of the target length. We can then calculate the fraction of J_{RMSE} due to the ballistic phase, compared to the stabilisation phase, as

$$J_{RMSE,rel} = \sqrt{\frac{\sum_i^{N_T} (l_{\text{ag},i} - l_{\text{ag,target}})^2 + (l_{\text{ant},i} - l_{\text{ant,target}})^2}{J_{SSE}}} \quad (22)$$

where N_T is the timestep corresponding to T_T.

The fraction of J_{RMSE} accrued during the ballistic phase is never less than 99%. This indicates that the performance metric is largely driven by the speed with which the simulation can reach the target (S1 Fig)

We next define time to stabilisation (T_S) as the first timepoint where the instantaneous muscle strain rate is less than 1% of maximum, and the muscle length is within $0.01 l_0$ of target. S2 Fig shows the correlation between T_T and T_S to RMSE. There is a strong positive correlation between both time metrics and J_{RMSE}, indicating that, in general, performance improves when time to target and time to stabilisation is lower. Note that all trials reach the target and achieve stabilisation within the allotted 0.4 s.

S3 Fig shows correlations between T_T, T_S, J_{RMSE} and mean coactivation through each simulation. Coactivation is strongly negatively correlated with each of these measurements. This indicates that, counterintuitively, trials that were fast and stabilized rapidly tended to exhibit higher levels of coactivation overall than trials that were relatively slow.
