# Supplementary material for: How muscle ageing affects rapid goal-directed movement: mechanistic insights from a simple model
Source: PLoS Comput Biol. 2026 Apr 15;22(4):e1014023. doi: 10.1371/journal.pcbi.1014023 (PMC13082658; doi:10.1371/journal.pcbi.1014023)
Supplement: Attachment — Submitted filename: Response to Reviewers.pdf [file pcbi.1014023.s006.pdf]

Response to Reviewers

We thank the reviewers for their thoughtful recommendations. We have responded to each point below (our responses in **bold**), revised the manuscript where appropriate, and believe the paper is improved as a result. Unless otherwise stated, line numbers in our responses refer to the revised manuscript with tracked changes.

Reviewer 1

In this paper the authors simplified the model described in Murtola and Richards (2022, 2023) to only include movements over the elbow joint. This single joint model was used to study a variety of parameters related to muscle activity and aging. The parameters that were examined and altered during the experiment were peak active force, maximum strain rate, activation time, deactivation time and stiffness. The parameter settings were selected based on previous knowledge and a thorough literature sweep. The simplified model applied was selected to enable us to explore the effects of varying parameters in a well-defined control task without having to analyze actual human data, which is noisy. They found four main terms (The Intercept term, Activation time, Maximum strain rate and Peak isometric force) and one interaction term (deactivation x stiffness) to be significant. Showing how a simplified model can be used to study specific parameters with settings representing different "ages". In addition, they show that co-contractions might not only be a result of perturbations in older people but a result of optimizing performance when movement is constrained the way it is in older people.

Data code and availability: Checked the link at it works, it does.

Abstract: I am missing the information that the model was created to represent movement over the elbow joint. Please include this information in the abstract.

We have replaced "…simplified model of a single joint…" with "…simplified model of *an elbow* joint…"

Introduction: I like the introduction and think that you have structured it nicely.

Thank you for this supportive feedback

Method:
- Line 67-68: Please specify which joint you are working with, in order to make it easier to follow. You could just put it in parenthesis, like this: ….rotational joint (the elbow joint) from one…

We have implemented the reviewer's suggested addition, as well as a clarifying sentence at the end of this paragraph: "Although we choose model parameters to approximate a human elbow joint, the model could be easily extended to other single-degree-of-freedom joints through judicious selection of parameters."

For the following reviewer comments, there may be some confusion regarding the methodological design. This may have stemmed from our presentation of the methodology, which we have amended (details to follow).

To clarify, we have swapped the presentation order of the methods (trajectory optimisation first, then parameter selection), and added a summary paragraph near the beginning of the methods section to explain our approach (line 64 in tracked changes manuscript):

"We first define the point-to-point movement as an optimal control problem (OCP). Using the above elbow model, we discretise and solve this OCP for a given set of parameters. The parameters of interest are activation and deactivation time constants (α_a, α_d), peak isometric force (F_{max}), maximum contraction velocity (v_{max}), and passive stiffness (c_1). We compare kinematic and excitation predictions from a baseline parameter set (from [34]) to empirical data of healthy elbow motion as a validation. Next, we choose parameter ranges based on

ageing literature to explore the effects of variation. We then perform full factorial parameter sweeps, solving the optimal control problem for every combination of parameter values over all their ranges simultaneously. This generates a dataset of performance metrics as a response with parameter values as independent predictors. To explore the relationships between these predictors, their interactions, and performance, we regress the data set onto a linear model including first-order interaction terms, and determine which parameters (or their interactions) have the strongest effect on performance."

• Line 139-159: The method selected here implies that you assume the relationship between the parameters to be independent and linear. I would be more confident about the results if the data was fitted to a non-linear multivariate regression model as well, since this would allow for more complex relationships. When working with purely isometric contractions the linear models have been enough to describe e.g., Force and EMG activity, but when moving into more naturalistic movement non-linear models have become required to describe the data. When looking at the supplementary correlation plots there also seems to be a simplification to call some of these relationships linear.

We thank the reviewer for pointing out where we have not been clear. The underlying parameters (activation and deactivation constants, muscle Fmax, Vmax, and stiffness) are independent by design, in the sense that they can be uniquely manipulated without affecting the value of other parameters. When we regress the performance landscape onto the linear model, we do not assume that parameters have independent effects on the performance, and include interaction terms. It turns out that the interactions are not very strong compared to independent terms (see Table 3), and that the linear model describes the simulated performance landscape very well (R^2 > 0.9). Both these findings surprised us, but the strong correlation between the fitted linear model and nonlinear simulated performance landscape justifies the choice of a linear model *post hoc*. To clarify, we have modified the text as follows (line 156):

> **"In order to determine which of the parameters, or their interactions, contribute to changes in performance in *the dataset generated by the parameter sweep simulations*, we performed linear regression of J_{RMSE} against the five muscle parameters and their interactions."**

• You apply a method where you change one parameter at a time. This has been shown to mask true performance optimum. In addition, it fails to account for interactions between parameters, which you clearly state that you assume that there isn't. However, another way to solve this would be by applying design of experiment and altering the parameters in a designed fashion. Even in a simple system I would assume the studied factors to be interdependent and have a complex correlation pattern. I suggest that you apply a different linear regression method that allow for the factors to be studied simultaneously.

Again, we apologise for not being clear. To clarify, we used two methodological designs: the simulation study design, and the linear regression model. For the simulation study, we explicitly test each parameter variation with respect to all other parameter variations in every combination (a full factorial design). This allows us to test the effect of isolated parameter changes, as well as their interactions. The simulation framework generates a data set (i.e. makes predictions about behavioural or performance changes as a function of parameter changes) but it is not necessarily a framework for analysing this dataset (i.e. determining which parameter changes have the largest effect).

We use the linear regression model to highlight the effects and interactions already present in the simulated dataset. The linear regression tests all first-order interactions under the linear model, and uncovers the dominant parameters determining output performance.

We believe we have caused confusion because of the order of presentation of the methods. We hope that reordering the presentation and the new summary paragraph (see above) have cleared up any misunderstanding.

• Model significance is not specified. You have not specified how you evaluate the quality of the models. Please add aa segment to the method describing how you made sure that the models were significant.

Though we agree that significance is an important parameter for statistical analyses, we disagree that it is relevant in our specific case. To expand on our response above, model significance is not relevant in the present study, since regression was used as a *dimensional reduction technique* rather than to test a particular hypothesis with uncertain data. Indeed, our data have little uncertainty- the optimisation procedure and simulations are deterministic, and there is no reason to expect nonlinear variation to follow a normal distribution.

For these reasons, we keep R^2 as it may indicate the degree to which the linearisation captures the underlying (nonlinear) variation in the simulated data, but we believe including *p* values or discussing statistical significance is not appropriate and risks confusing readers about the motivation of the regression step.

This question may stem from a misunderstanding of our approach and the goal of the applied regression. We hope the summary paragraph (see above) clears up this confusion.

Results:

• Figure 2: Were the empirical data preloaded in any way? Were the simulations loaded in a similar fashion? Does this simulation represent the baseline conditions?

The empirical data were not preloaded, according to Forgaard et al. 2013, and the simulations match this condition. The simulation does indeed use baseline parameters as stated in the caption title; however, the target excursion is 60 degrees in both the empirical and simulated data here, rather than 90 degrees in the remainder of the simulated data. We have added a clarifying final statement to the figure caption.

Figure 5: RMSE varies between 0.07 and 0.0735. How significant is this difference? Interpreting this as "increasing stiffness decreases performance" sounds like a stretch, these are marginal differences highlighted by changes in color. Please provide the rational for how such a small increase in RMSE is indicative of "increasing stiffness decreases performance" (line 199). Even when taking into account that you have standardized your data to have a standard deviation =1, this seems small.

The reviewer raises a good point. Over the entire parameter space (Figure 4), the RMSE varies from 0.0534 to 0.0943 (we have added this information to the Figure 4 caption). In figure 3, the RMSE varies by $0.0043l_0$, corresponding to 10.6% of the maximum possible performance change in the dataset. In our view, this is appreciable and indicates a 17% increase in time to target. In any case, we have now made this information explicit in the caption to figure 5 and on line 232 of the track changes document, so readers may make their own judgements.

• How do you evaluate the significance of the main model where you obtain Table 3?

See our above explanation regarding how significance is not a relevant metric in the present study.

• Line 235-242 You mention R2, but not Q2? It is indeed important to have a well-fitted model, but also to have it explain how well your model predicts data. Why is not Q2 reported? Why is R2 (and Q2) not reported for your main model?

If we were extrapolating these results to an external empirical dataset, a Q2 stat would indeed be an important metric. However, as explained above, the linearisation is used to simplify the *simulated* dataset, and to look for patterns therein, and is not meant to exactly predict empirical patterns.

Indeed, the "main model" (the simplified single-joint model subjected to optimisation) is meant to replicate broad patterns of rapid motion control (e.g. triphasic activation, cocontraction), but not to replicate it exactly (as Figure 2 demonstrates). The performance landscape (and the simplified linearisation thereof), in our view, captures general qualitative features of performance as a function of these physiological parameters (which themselves are approximations of more complex muscle properties), but should not be interpreted as a precise quantitative predictor. We report R^2 for the linearisation to indicate how well it captures a performance landscape arising from nonlinear dynamics, but we do not expect it to match empirical reference data precisely. Moreover, such a dataset does not exist, as far as we are aware.

We have clarified the limitations of our approach in the text:

Beginning of discussion:
***"While the simulation is not an exact simulacrum of human behaviour-- for example, the excitation peaks come later than empirical EMG signals--* the general match to empirical patterns *(with no fitting to data)* shows that the simplified model captures salient features of rapid targeted joint excursions."**

Limitations section:
"Altogether, the simplifications inherent in the modelling approach, and the deviations from empirical behaviour (particularly in EMG patterns) suggest caution in extrapolating quantitative findings to human data. However, the broad patterns (e.g. triphasic excitation, smooth reaching trajectories in realistic time) suggest that the model captures some qualitative features well. The simplicity of the model allows us to point to mechanistic links between physiological parameters and performance outcomes. These should be interpreted within the simplifying assumptions of the model itself, and their application to biological movement awaits confirmation from biological experimentation."

Discussion:
• Nicely structured and clear. I only miss one thing. You should include discussion about the timing of the empirical compared to simulations, where the simulation activity occurred much slower compared to the empirical (Figure 2). What is the impact of this on your results?

This is an important issue, and we thank the reviewer for pointing this out. We have now discussed this issue in the "limitations" section, starting from line 475 (tracked changes document)

Reviewer 2:

This manuscript investigated the effect of parameters for muscle contraction on a single joint reaching movement via a computer simulation. There results show that there is a non-trivial interaction between the stiffness of parallel elastic component and muscle deactivation rate, which is very interesting, However, I have some suggestions.

Introduction
The authors use a simplified reaching as a model movement. But, in Introduction, they discuss performance of locomotion and fall risk, which is more relevant to lower extremity. That seems to be a big stretch to me since mechanical demand imposed on arms during reaching is completely different from that on leg muscles during walking or running. The authors could focus on the argument mainly with reaching movement, which would be more natural to me.

We thank the reviewer for catching this important gap. We have expanded the sentence around a relevant cited study (line 12):

> **"Performance reductions in reaching tasks are associated with negative health outcomes, particularly fall risk. Older adults rely more on arm reactions for balance recovery, but are slower to initiate and execute reach-to-grasp [22]"**

Methods
Their model does not have a series elastic component (SEC). Due to SEC, the phase shift between the entire muscle length and fascicles can be significant in lower extremity during fast calf-raise movement (e.g. Takeshita et al. 2006), but maybe not so significant in forearm movement (Amis et al. 1987). While the justification seems to be discussed in the previous study, the authors should also provide their own justification.

References
λ J Appl Physiol. 2006 Jul;101(1):111-8. doi: 10.1152/japplphysiol.01084.2005. Epub 2006 Feb 23.

Resonance in the human medial gastrocnemius muscle during cyclic ankle bending exercise. Daisuke Takeshita 1, Akira Shibayama, Tetsuro Muraoka, Tadashi Muramatsu, Akinori Nagano, Tetsuo Fukunaga, Senshi Fukashiro
λ J Physiol 1987 Aug:389:37-44. doi: 10.1113/jphysiol.1987.sp016645. Relative displacements in muscle and tendon during human arm movements. A Amis 1, A Prochazka, D Short, P S Trend, A Ward

We thank the reviewer for pointing this out. We have now discussed some consequences for the exclusion of a SEC, and our rationale for it, in the limitations and future directions section (starting on line 492):

> **"While excitation and movement onset were coincident in the simulation, empirical EMG began rising about 50 ms before movement onset. This phenomenon, known as "electromechanical delay" (EMD) [66] arises from electrochemical and mechanical processes that show large inter-individual variation. One key contributor to EMD is tendon dynamics, which have not been modelled here. While the effect of ageing on tendon mechanics is complex and sometimes unclear [67], future studies could expand the performance landscape by incorporating tendon stiffness and viscoelastic effects."**

The authors should provide the properties of input signals. Is the value of input signals at each time bin determined or some parameterizations were used etc.

We apologise for not being clearer. The "Trajectory optimisation section" states that inputs are "time-varying excitation values that minimize the sum of squared position error (SSE) to a target. To clarify further, we have added an additional sentence to the methods:

> **"Excitation inputs were piecewise constant over these [0.5 ms] timesteps, constrained between [0,1] (but otherwise free to be selected by the optimisation routine)"**

Minor comments:
L 118: "(see Supplemental Information)" should refer to a particular section of the supplemental info.

We have amended the references to be more specific
